# Gastric lymphangioma: a case report and review of literature

**DOI:** 10.1186/s12876-022-02431-6

**Published:** 2022-09-04

**Authors:** Kunhao Bai, Yujun Dai, Chen Jiang, Shiyong Lin, Guobao Wang

**Affiliations:** 1 Department of Endoscopy, Sun Yat-Sen University Cancer Center, State Key Laboratory of Oncology in South China, Collaborative Innovation Center for Cancer Medicine, 651 Dongfengdong Street, Yuexiu District, Guangzhou, 510060 Guangdong China; 2 Department of Epidemiology, School of Public Health, Sun Yat-Sen University, Guangzhou, Guangdong China; 3 Department of Hematology, Sun Yat-Sen University Cancer Center, State Key Laboratory of Oncology in South China, Collaborative Innovation Center for Cancer Medicine, Guangzhou, Guangdong China; 4 Department of Pathology, Sun Yat-Sen University Cancer Center, State Key Laboratory of Oncology in South China, Collaborative Innovation Center for Cancer Medicine, Guangzhou, Guangdong China

**Keywords:** Gastric lymphangioma, Endoscopic ultrasonography, Subepithelial lesions, Case report

## Abstract

**Background:**

Gastric lymphangioma is one of the highly rare benign tumors characterized by multilocular or unilocular lymphatic spaces. Herein, we report a case of lymphangioma in the gastric antrum.

**Case presentation:**

A 77-year-old male patient who had been experiencing epigastric discomfort for a year was presented to our hospital. A gastric subepithelial lesion was diagnosed by upper endoscopy and was entirely excised via diatal subtotal gastrectomy. Endoscopic ultrasonography revealed an echoless homogenous echo pattern in the third wall layer. A lymphangioma was diagnosed by pathologic investigation of the resected specimen. The PubMed, Embase and Web of Science databases were reviewed for literature in English while using the keywords of “gastric lymphangioma” or “lymphangioma of stomach” or “gastric lymphatic cyst” or “lymphatic cyst of stomach” and the results were discussed.

**Conclusion:**

Gastric lymphangioma is a rarely occurring submucosal tumor that should be considered when diagnosing subepithelial lesions in the stomach.

## Background

Literature reports lymphangioma as a benign microcystic lymphovascular lesion located mostly in the axilla, groin, and neck and is characterised by dilated lymphatic channels [1]. The occurrence of lymphangioma in the stomach is not very common. This case reports a patient presented with epigastric discomfort. A broad-based, elevated subepithelial gastric lesion was diagnosed during a subsequent upper endoscopy. Endoscopic ultrasonography (EUS) revealed an echoless homogenous echo pattern in the third wall layer (submucosa) with well-defined inner septations. The evidence of lymphangioma was shown by the pathologic examination of the resected lesion. In the current case report, we evaluated the clinical symptoms, EUS signs, white-light endoscopic appearances, histopathologic features, and treatment course of this benign tumor, followed by a review of the relevant literature.

## Case presentation

**Fig. 1:**
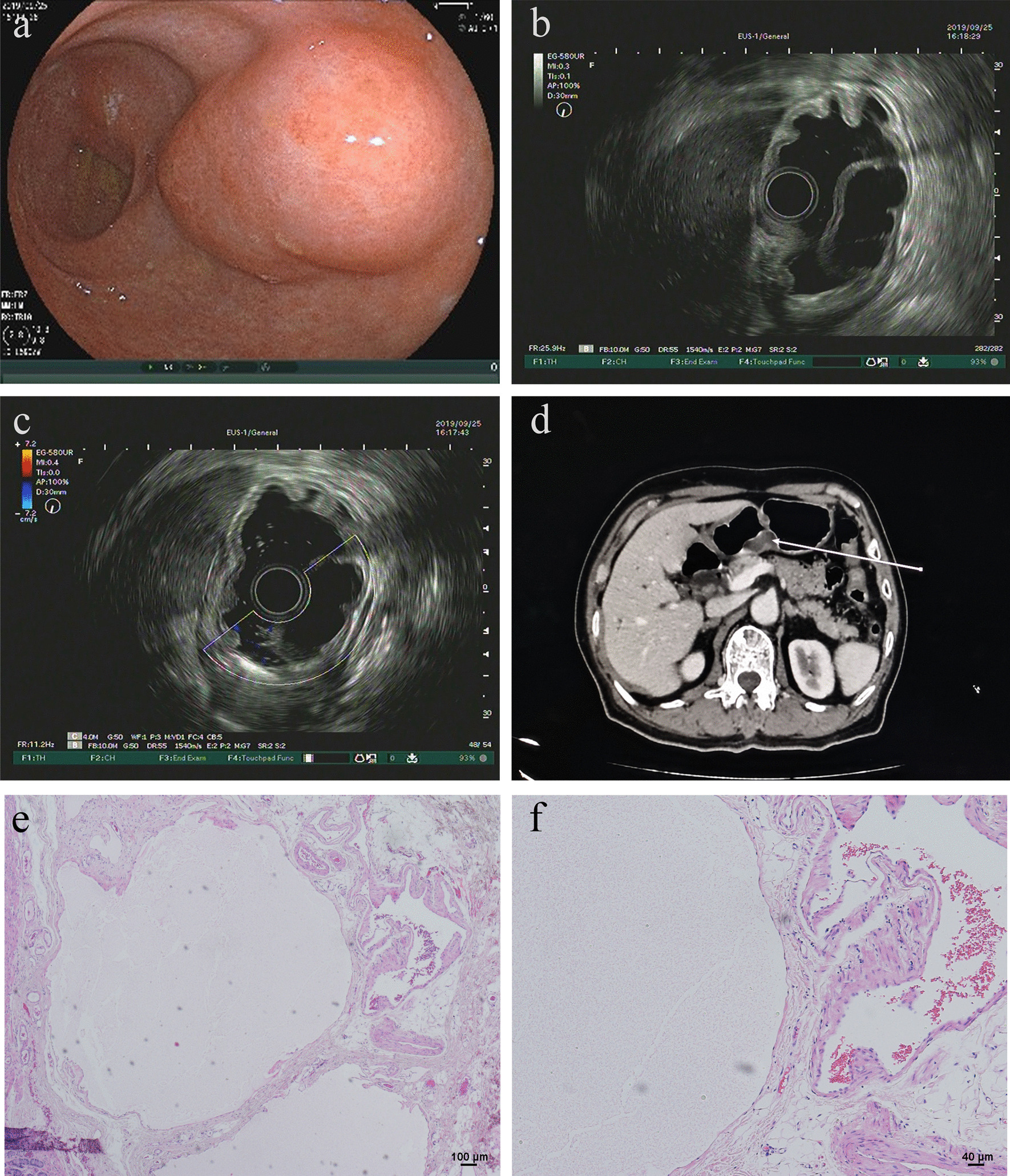
Diagnosis of gastric antrum lymphangioma. **a** The surface of the overlying mucosa was normal. **b** EUS revealed an homogeneous echoless mass with a high echogenic separation inside. **c** No signs of blood flow. **d** Abdominal contrast-enhanced CT scan revealed a low density nodular mass without apparent enhancement. **e** Histopathological examination showed thin-walled, dilated lymphatic channels separated by fine fibrous stroma (hematoxylin‑eosin staining; magnification, × 40). **f** High power histopathological view showed flat endothelial cells lined the lymphatic canals (hematoxylin‑eosin staining; magnification, × 100)

**Fig. 2:**
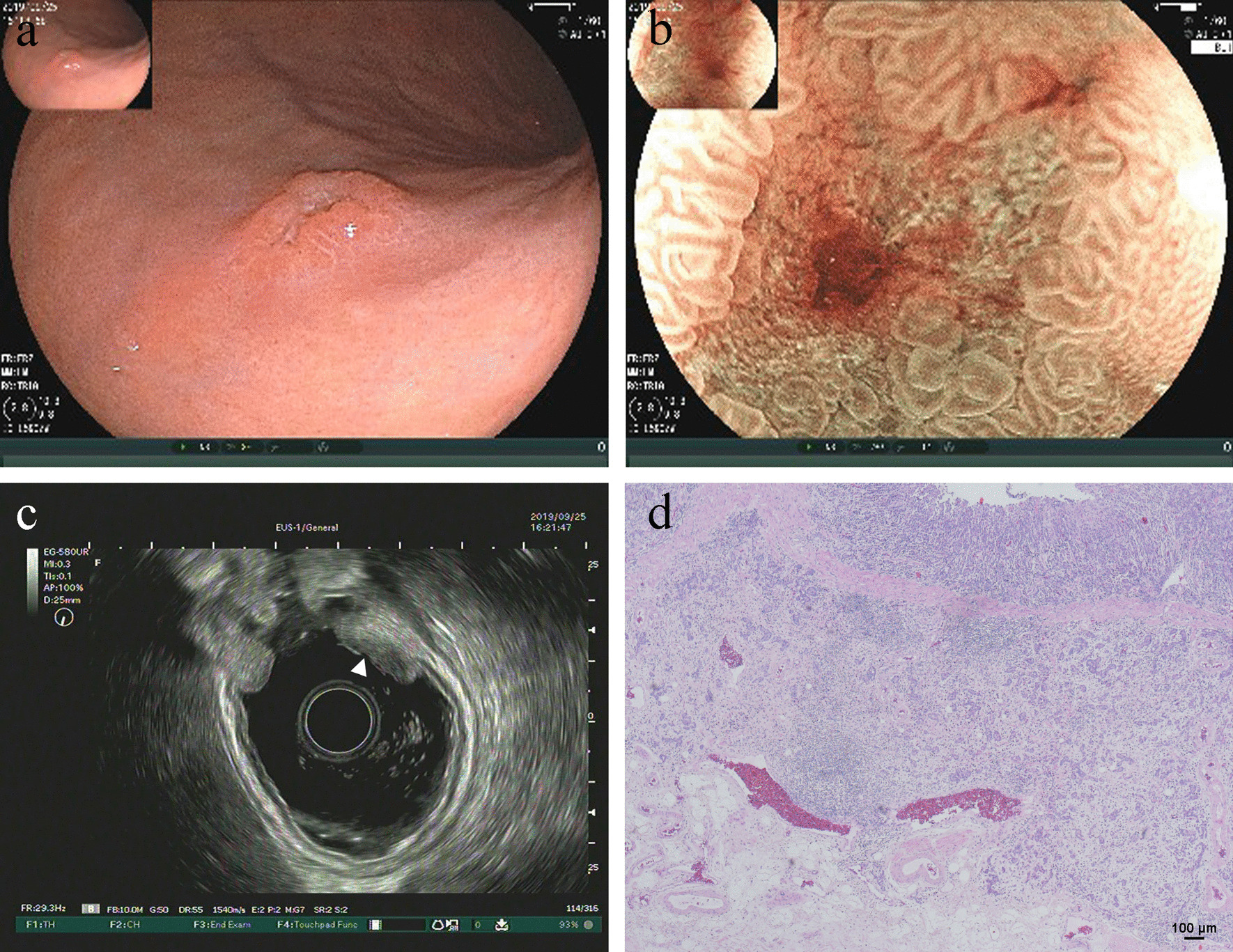
Diagnosis of gastric body carcinoma. **a** White-light endoscopy showed a slightly elevated (IIa) and depressed (IIc) lesion. **b** Magnifying endoscopy with blue laser imaging (ME-BLI) showed demarcation line (DL), absent microsurface pattern (AMSP), and irregular microvascular pattern (IMVP). **c** EUS revealed a hypoechoic lesion infiltrating into the third layer. **d** Histopathological examination showed carcinoma cells infiltrating into the submucosa (hematoxylin‑eosin staining; magnification, × 40)

A 77-year-old man complained of epigastric discomfort for almost one year. He had an esophagogastroduodenoscopy in our hospital in September 2019 and a gastric subepithelial lesion (SEL) on the posterior wall of the stomach antrum was diagnosed. The lesion was sessile, broad-based and elevated in size, measuring 3.0 cm in diameter during white-light endoscopy. The surface of the overlying mucosa was red–orange normal stomach mucosa with no erosion or ulcer (Fig. 1a). Further endoscopic assessment of the gastric lesion with EUS revealed an homogeneous echoless mass with a high echogenic separation inside (Fig. 1b). Moreover, there were no signs of blood flow (Fig. 1c). The lesion was mostly in the submucosal area, and it didn't impact the underlying gastric muscularis propria. Abdominal contrast-enhanced computed tomography scan revealed a low density nodular mass located in the gastric antrum, without apparent enhancement (Fig. 1d). Because of the conjunction of gastric body adenocarcinoma (Fig. 2), this submucosal tumor was evaluated clinically as a benign lesion that was entirely excised by diatal subtotal gastrectomy.

The size of the resected lesion was 2.5 × 2.0 × 1.0 cm. The lesion was serially sectioned after routine formalin fixation to reveal a soft, whitish-grey, and slightly spongy cut surface. There was no solid nodule or tumor found. Further, there was no evidence of bleeding or necrosis. When observed under a microscope, the lesion mainly affected the submucosa layer and was composed of thin-walled, dilated lymphatic channels separated by fine fibrous stroma (Fig. 1e). Flat endothelial cells lined the lymphatic canals (Fig. 1f), and the overlaying epithelium or tumor had no malignancy or dysplasia. All microscopy images was captured by light microscope (Olympus BX53, Japan) with an attached Olympus cellSens Standard software.

The patient's stay in the hospital after surgery went smoothly. He was well during his 6-month follow-up and had no problems. All procedures performed in this study involving human participants were in accordance with the 1964 Helsinki Declaration and subsequent relevant ethics. Written informed consent was obtained from the patient.

## Discussion and conclusions

Lymphangioma is a rare benign soft tissue tumor that affects the gastrointestinal tract in adults. About 1% of lymphangiomas originate in the gastrointestinal tract, where the colon is the most common site for lymphangiomas [2]. Only 24 cases of gastric lymphangioma published in English were found in the PubMed, Embase and Web of Science databases. The major advantage for discussion of this case is that we provided the fairly typical and detailed case images, including white-light endoscopic appearances, EUS signs, contrast-enhanced CT images and histopathological images. These images could help clinicans to get a better understanding of this rare benign tumor.

The characteristics of the 24 cases are shown in Table 1. The median age is 51.5 years, and 57.1% (12/21) is male. In most cases, gastric lymphangioma is a single tumor. Although tumors can range in size from 1.2 to 44 cm, most are less than 5 cm in diameter (65.0%,13/20), with median diameter of 3.8 cm. The tumor is most commonly found in the gastric antrum (36.3%,8/22). There is only one case that is located in the gastric cardia [3].

**Table 1:** Cases of Gastric Lymphangioma in the English Literature

Case	Author	Year	Country	Age	Gender	Size (cm)	Location	Surface	Layer	Septation	EUS	Chief complains	Treatment
1	Chadock P [10]	1964	USA	63	Male	5	Gastric body	Normal	Submucosal	NA	NA	acUte gastroenteritis	Surgery
2	Ochsner SF [11]	1965	USA	NA	NA	NA	NA	NA	Submucosal	NA	NA	NA	Surgery
3	Fleming MP [12]	1970	USA	79	Male	3.5	Lesser gastric curvature	Normal	Submucosal	NA	NA	Epigastric discomfort	Surgery
4		1970	USA	NA	NA	NA	NA	NA	Submucosal	NA	NA	NA	Surgery
5	Isaacson R [13]	1970	USA	NA	NA	NA	NA	NA	NA	NA	NA	NA	NA
6	Drago JR [5]	1976	USA	5	Feamle	7	Gastric body	Hemorrhagic	NA	NA	NA	Traumatic rupture	Surgery
7	Colizza S [4]	1981	Italy	58	Female	6	Gastric antrum	Hemorrhagic	Lamina propria and submucosal	NA	NA	Hematemesis and melena	Surgery
8	Yamaguchi K [14]	1989	Japan	66	Male	2.5	Gastric antrum	Normal	Submucosal	NA	NA	Epigastric discomfort	Surgery
9	Kim YJ [15]	1989	Korea	46	Male	3	Gastric antrum	Normal	Submucosal	NA	NA	Asymptomatic	Surgery
10	Hizawa K [9]	1996	Japan	46	Female	1.2	Gasric body	Normal	Lamina propria and submucosal	Yes	Medium-echo	Asymptomatic	EMR
11		1996	Japan	43	Female	3.6	Gastric antrum	Normal	Submucosal	Yes	Anechoic	Epigastric discomfort	EMR
12	Tsai CY [16]	1997	China	58	Female	1.5	Gastric antrum	Normal	Submucosal	Yes	Anechoic	Asymptomatic	Surgery
13	Park SJ [17]	1999	Korea	NA	NA	3.5	Gastric angle	Normal	Submucosal	NA	NA	Epigastric discomfort	Surgery
14	Ishikawa N [18]	2001	Japan	57	Male	1.5	Gastric Angle	Normal	Submucosal	Yes	Anechoic	Asymptomatic	EMR
15	Kim HS [19]	2001	Korea	68	Female	2.5	distal gastric body	Normal	Submucosal	Yes	Anechoic	Epigastric discomfort	EMR
16	Gockel I [20]	2001	Germany	16	Male	2.20 kg	Lesser gastric curvature	Normal	NA	Yes	NA	Abdomianl mass	Surgery
17	Das CJ [21]	2006	India	17	Male	NA	Gastric body and antrum	NA	Submucosal	Yes	NA	Epigastric discomfort	Surgery
18	Leland HA [22]	2011	USA	16	Female	12	Gastric body	Normal	NA	Yes	NA	Abdomianl mass	Surgery
19	van Oudheusden TR [23]	2013	Netherlands	44	Male	44	Gastric body	NA	NA	Yes	NA	Rapid abdominal distension	Surgery
20	Chen G [3]	2016	China	18	Female	4	Gastric cardia	Normal	Submucosal	Yes	Medium-echo	Epigastric discomfort	ESD
21	Zhuang KM [24]	2019	China	24	Female	22	Gastric body	NA	NA	Yes	NA	Epigastric discomfort	Surgery
22	Matsushita A [8]	2020	Japan	76	Male	4	Distal gastric body	Normal	Submucosal	Yes	NA	Constipation	Surgery
23	Nayak M [6]	2020	India	65	Male	6	Gastric antrum	Normal	Submucosal and muscularis propria	Yes	NA	Gastric outlet obstruction	Surgery
24	Ali HA [7]	2021	Morocco	21	Male	25	Lesser gastric curvature	Perforation	NA	Yes	NA	Perforation	Surgery
25	Present case	2021	China	77	Male	3	Gastric antrum	Normal	Submucosal	Yes	Anechoic	Epigastric discomfort	Surgery

*USA* United States of America, *EMR* endoscopic mucosal resection, *ESD* endoscopic submucosal dissection

The clinical manifestations of gastric lymphangioma are generally nonspecific. Epigastric discomfort, as shown in our case, is the most common complaints (40.9%, 9/22). They may be asymptomatic and can be identified incidentally (18.2%, 4/22). Sometimes, they may have various chief complaints, even could cause gastrointestinal hemorrhage [4], tumor rupture [5], gastric outlet obstruction [6] or gastric perforation [7], depending upon the size and location of a tumor. There are only two cases that gastric lymphangioma coexists with mucosal gastric cancer. In one case [8], the mucosal adenocarcinoma is located immediately above the lymphangioma.While in our case, the gastric antrum lymphangioma and the gastric body adenocarcinoma are located apart.

Under white light endoscopy, the tumour is red–orange typical gastric mucosa with no ulcer or erosion; it is pliable upon compression with biopsy forceps. A large tumor may be translucent and lustrous. The depth and size of a lesion could be assessed with EUS. Under EUS, a cystic homogeneous echoless mass with hyperechoic inner septations in the third layer is the hallmark appearance of a gastric lymphangioma. The use of EUS examination can assist distinguish lymphangioma from gastric GIST (gastrointestinal stromal tumor), the most common gastric SEL. However, in the two cases composed of multiple small lymphatic spaces [3, 9], EUS could reveal a homogeneous medium echogenic mass.

Microscopically, gastric lymphangioma is characterized by localized proliferation of thin-walled, dilated lymphatic channels of diverse diameters observed in the current case. In the overlying squamous epithelium, there is no dysplasia. In most cases, determining the accurate diagnosis based on histological findings is not difficult. A negative immunoreactivity for FVIII, positive immunostaining pattern in lymphatic endothelial cells for D2-40 could help establish the diagnosis of lymphangioma in cases where it has to be distinguished from hemangioma.

Depending on the size of the tumor, different treatment options for gastric lymphangioma may be used. A large symptomatic tumor can be surgically removed. Endoscopic therapy was previously not used to resect gastric lymphangiomas. Kazuoki Hizawa et al. [9] firstly reported two cases of gastric lymphangioma resected by endoscopic mucosal resection (EMR) in 1996. Further more, the large tumor up to 4 cm in size has been reported to be totally and successfully resected by endoscopic submucosal dissection (ESD) without severe adverse consequences in 2016 [3], thanks to advances in endoscopic procedures and the accumulation of surgical expertise by endoscopists. Endoscopic resection has been the treatment of choice for gastric lymphangioma, offering benefits over surgery in terms of minimal injury, elevated efficacy and safety, and improved quality of life following resection.

To summarize, gastric lymphangioma is a rare submucosal tumor that should be considered when diagnosing gastric SEL. The use of EUS for preoperative diagnosis and evaluation of tumor size and depth is critical.

## Data Availability

All the data regarding the case are available within the manuscript.

## Abbreviations

EUS: Endoscopic ultrasonography
SEL: Subepithelial lesion
EMR: Endoscopic mucosal resection
ESD: Endoscopic submucosal dissection
ME-BLI: Magnifying endoscopy with blue laser imaging
AMSP: Absent microsurface pattern
IMVP: Irregular microvascular pattern
